# Investigating the effect of limited spectral information on NIRS-derived changes in hemoglobin and cytochrome-c-oxidase concentration with a diffusion-based model

**DOI:** 10.1364/BOE.531775

**Published:** 2024-09-17

**Authors:** Georgina Leadley, Robert J. Cooper, Topun Austin, Jeremy C. Hebden, Gemma Bale

**Affiliations:** 1 Department of Paediatrics, University of Cambridge, UK; 2 Department of Engineering, University of Cambridge, UK; 3 Department of Medical Physics and Biomedical Engineering, University College London, UK; 4 Department of Physics, University of Cambridge, UK

## Abstract

This paper investigates the theoretical capability of near-infrared spectroscopy (NIRS) systems to accurately measure changes in the oxidation state of cerebral cytochrome-c-oxidase (CCO) alongside the hemoglobins, for a deeper understanding of NIRS limitations. Concentration changes of oxy and deoxyhemoglobin (HbO and HbR) indicate the oxygen status of blood vessels and correlate with several other physiological parameters across different pathologies. The oxidation state of CCO indicates cellular energy usage efficiency through oxidative metabolism, potentially serving as a biomarker for brain and other tissue disorders. This study employs an analytical model based on the diffusion equation and statistical analyses to explore the dependency of estimated concentration changes on various systematic parameters, such as choice of wavelengths, spectral bandwidth, and uncertainties in extinction coefficient (*ε*) and differential pathlength factor (DPF). When there is a 10% uncertainty in DPF and *ε*, errors were found to be highly dependent on the number of discrete wavelengths, but not on their bandwidth if appropriate considerations are taken to account for it.

## 1. Introduction

The oxidation state of CCO is an indicator of the efficiency of energy usage and metabolism of cells [1] as it is the terminal electron acceptor in the electron transport chain. As a biomarker for metabolism it can provide insight into the energy dynamics of the brain and other organs, so has promise as a valuable signal, particularly in critical care settings. Near-infrared spectroscopy (NIRS) can measure changes in the concentration of oxidized CCO (oxCCO) as its absorption spectrum is dependent on its redox state. However, the measurement of changes in the concentration of oxCCO is not straightforward, as its concentration is an order of magnitude smaller than those of oxy and deoxyhemoglobin (HbO and HbR) in tissue. Additionally, its absorption spectrum in the near-infrared is relatively featureless [2,3]. Nevertheless, many studies have been undertaken correlating the NIRS-derived oxCCO signal with physiological parameters [2,3].

The concentration of oxCCO has been shown to change when the brain is under oxidative stress in clinical studies in both critically ill newborns and adults [4–6]. However, changes in concentration of oxCCO show a varied response to similar functional paradigms across several studies [2,3,7]. While the underlying mechanism of CCO redox changes is understood, the behaviour of oxCCO in a healthy brain as observed by NIRS techniques has not been fully characterised and results are difficult to interpret. Some recent studies aiming to investigate the response of the adult pre-frontal cortex to cognitive processing and attention tasks have yielded changes in oxCCO that are opposite in direction [7–10]. The inconsistency in the observed changes could be due to the different hardware or processing techniques used, or due to the different cognitive tasks employed. Additionally, studies quantifying oxCCO changes in muscle have also obtained opposing results depending on whether the system used in the study employed a broadband source or several discrete wavelengths [10–15].

NIRS instrumentation can be divided into two principal categories: broadband and discrete wavelength. Broadband systems commonly employ a filtered white light source for illumination and collect the diffusely transmitted light using fibre-optic probes coupled to a spectrometer which measures the NIR spectrum. Conversely, discrete wavelength systems use sources such as LEDs or laser diodes to selectively illuminate at specific NIR wavelengths. These systems typically employ small discrete detectors such as photodiodes or single-photon avalanche detectors.

While discrete wavelength systems confer the advantage of enhanced wearability relative to broadband systems, there exists a potential compromise in terms of chromophore concentration quantification accuracy. Despite the assumption of precision in quantifying concentration changes in HbO and HbR with discrete wavelength systems [16,17], their efficacy in capturing changes in oxCCO has not been explored to the same extent.

The motivation for this study was threefold. Some previous simulation and phantom works have shown that there are severe implications for small uncertainties in measurement parameters [18,19]. There have been no consistent efforts to determine the accuracy of extinction coefficients over the last 20 years, with oxCCO never measured in realistic temperature environments. The likely errors in NIRS measurements due to the multiple factors of uncertainties in DPF and *ε*, number of measurement wavelengths and their bandwidth have not been investigated simultaneously. Were the likely measurement errors known with greater accuracy, future system design could take into account the intended use according to these simulations and proceed accordingly.

This work focuses on theoretical experiments in the quantification of changes in HbO, HbR and oxCCO. NIRS systems designed for deployment in brain research are traditionally constructed and then tested on phantoms or a handful of subjects, with hardware selected based either on that of previous systems or the findings from historic algorithmic studies. Past efforts to quantify optimal NIRS parameters include works employing genetic algorithms, Monte Carlo simulations and phantom measurements [19–22]. A 2015 study employed a genetic algorithm to determine the minimum number of wavelengths required for a system to resolve changes in oxCCO concentration, with results pointing towards a combination of at least 5 wavelengths which evenly span the 780-900 nm spectral rang [21]. It has been postulated that exclusion of shorter wavelengths could improve the resolution of oxCCO by lessening the contribution from other types of cytochrome which also absorb in the near-infrared rang [23]. Simulation work by Truong et al. [19] found that the variance and uncertainty of concentration change measurements was much higher when using only a small number of wavelengths when compared to a broadband range, even for a high signal-to-noise ratio (SNR). Exploration of the effects of using different numbers of measurement wavelengths of varying bandwidth, different combinations of wavelengths and errors in other parameters has not been performed with diffusion simulations as described in this paper.

As a consequence of the challenges in measuring oxCCO, any errors in assumptions made in calculating concentration changes can easily be amplified, with the number and specific values of wavelengths chosen being significant potential sources of error. A simulation based on the diffusion approximation describes photon migration through media using different wavelengths, and can be used to calculate concentration change errors. By leveraging these realistic simulations, measurement errors can be explored, quantified and potentially minimised for future studies.

The aim of this study is to explore the theoretical conditions necessary to observe a genuine oxCCO signal using NIRS and what assumptions in the measurement of the signal can result in errors. The specific objectives for this paper were to:

- Quantify the measurement errors in NIRS-derived HbO, HbR and oxCCO concentration changes using a diffusion-based simulation.
- Determine the effect of uncertainties in parameters used to calculate concentration changes such as extinction coefficient and differential pathlength factor on measurement errors.
- Explore the effects of using different numbers of wavelengths and different combinations of wavelengths on these measurement errors.
- Investigate the effect of increasing bandwidth of discrete wavelength sources on measurement errors.
- Identify an ideal SNR threshold for NIRS measurements using broadband and discrete wavelength systems.

## 2. Methods

Construction of a simulation using a diffusion-based model allows assessment of the likely errors associated with different hardware and software elements of NIRS equipment. The definition of hardware elements are design choices such as number and combination of measurement wavelengths and source type. Software elements refer to the chosen extinction coefficients and DPF values used to calculate changes in chromophore concentrations, and methods for accounting for spectral bandwidth. A known concentration change is induced in a finite homogeneous slab, and the changes in optical parameters calculated. These parameters can then be used to estimate the concentration changes using the same processing pipeline as conventional NIRS equipment. The estimated concentration change is then compared to the known change, and the percentage error between the two values is determined.

As the wavelength-specific model parameters can be controlled, this allows the errors in chromophore concentrations associated with any parameter changes to be isolated and estimated. Parameters that are changed include:

- the number of measurement wavelengths used
- the bandwidth of the sources used as input to the diffusion equation
- the SNR of the detected output from the diffusion simulation
- the values of extinction coefficient and DPF used to calculate chromophore concentration changes

### 2.1. Diffusion simulations

The diffusion approximation to the radiative transfer equation (RTE) is commonly used to describe photon transport in a highly scattering medium such as biological tissue, and we have employed a diffusion-based model described by Contini et a [24]. An input absorption and scattering coefficient for each chromophore and wavelength of interest is used to simulate photon migration through a homogenous slab geometry to produce values of wavelength-dependent diffuse reflectance and DPF for a set source-detector distance. These values are then used as input to the modified Beer-Lambert law (MBLL) (Eq. (7)) which estimates the change in concentration of each chromophore to simulate NIRS experiments. The model used for this simulation is an analytical solution to the time-dependent diffusion equation for a finite slab of given thicknes [24]. For these simulations, the slab thickness was set to 50 mm, the source-detector separation set to 30 mm and the refractive index of the slab 1.4. The input parameters to the model are shown in Table 1.

**Table 1.** Input concentrations to diffusion simulation. Base concentrations were varied within three separate limits. The concentration changes in Model 1 were informed by the average changes seen in infant functional studies as estimated from papers in a recent review article [3]. Model 2 was informed by the average changes in adult studies from the same review. Model 3 reflects the expected values from phantom experiments which can be much larger [25].

Parameter	Base Concentration (μM)	Max Concentration Change (μM)		
		Model 1 (eg. neonate)	Model 2 (eg. adult)	Model 3 (eg. phantom)
HbO concentration	56	± 1.5	± 6.0	± 10.0
HbR concentration	24	± 1.0	± 4.0	± 7.0
oxCCO concentration	4.9	± 0.3	± 1.5	± 3.0

The input absorption coefficients $\mu_{a}$ are generated using pre-specified chromophore concentrations (*c*) using the extinction coefficients (*ε*) of each chromophore of interest (*k*) at each measurement wavelength ( λ ) using:

(1) $$\mu_{a}(\lambda )=\sum_{k}\varepsilon_{k}c_{k}$$

where the extinction coefficients *ε* used are taken from the Homer2 software package (J.M. Schmitt,1986). The reduced scattering coefficient $\mu_{s}^{\prime }$ is then calculated using the following equation, where it has been normalised to a value of 1.0 mm^−1^ at 800 nm [26–28]:

(2) $$\mu_{s}^{\prime }(\lambda )={\left(\frac{\lambda }{800(nm)}\right)}^{-1.2}$$

These values of wavelength-dependent absorption and reduced scattering coefficients are then input into the equations outlined in Contini et al. [24], which return values of diffuse reflectance and DPF for the initial and changed concentration values.

Broadband, continuous-wave systems can select wavelengths at successive 1 nm intervals and the diffuse reflectance generated via diffusion simulations needs no modification when input to the modified Beer-Lambert law.

For NIRS systems employing a small number of discrete wavelengths, separate considerations must then be made within the theory according to the spectral shape and bandwidth of the sources. Two cases are considered for the diffusion simulations: one where the spectral bandwidth is accounted for via modifying the extinction coefficients used, and the other where the extinction coefficient at the central wavelength is used without considering the source bandwidth. For the first case, we modify the extinction coefficients based on the spectral profile of the source and the diffuse reflectance re-calculated using the diffusion approximation. The shape of the broadband output is first modified according to the characteristics of the discrete wavelength sources. An estimation for the emission profile of a diode source can be given by the Lorentzian distribution, which can be used to model the spectral profile $p_{k}(\lambda )$ of systems using *k* discrete sources with central wavelength *λ_k_* and can be described using their full-width half maximum (*w*).

(3) $$p_{k}(\lambda )=\frac{1}{\pi }.\frac{\frac{w}{2}}{{\left(\lambda -\lambda_{k}\right)}^{2}+{\left(\frac{w}{2}\right)}^{2}}$$

The spectral profile of the source can then be used to determine the effective extinction coefficient $\varepsilon_{n}^{\prime }(\lambda_{k})$ of each chromophore at each wavelength of interest. The effective intensity at each discrete wavelength can also be calculated by integrating the intensity over the spectral profile of each LED source:

(4) $$\varepsilon_{k}^{\prime }(\lambda )=\sum \varepsilon_{n}(\lambda ).p_{k}(\lambda )$$

(5) $$I_{k}^{\prime }(\lambda )=\int I(\lambda )(\lambda )d\lambda$$

The effective intensity, extinction coefficient, and the reduced scattering coefficient at each wavelength are adjusted for a normalised intensity and again used as input to Contini’s equations, resulting in an output diffuse reflectance and DPF value for the specified change in concentration. These values can then be used to calculate the change in concentration of each chromophore of interest using the modified Beer-Lambert law. NIRS conventionally relies on measuring changes in intensity under conditions where the source intensity, the unknown coupling loss and the unknown scatter-dependent geometric factor remain constant, i.e.

(6) $$\Delta \;ln\;I=-\Delta \mu_{a}.\beta d=-\beta d\underset{k}{\sum }\Delta c_{k}\varepsilon_{k}$$

where β represents the differential pathlength factor (DPF) and d represents the source-detector separation. The DPF is introduced to the equation to account for light scattering in tissue. The modified Beer-Lambert law (MBLL) describes the method of calculating the resulting concentration change using the change in attenuation (ΔA) and extinction coefficients at each measurement wavelength and is given by:

(7) $$\left[\begin{array}{c} \Delta HbO\\ \Delta HbR\\ \Delta oxCCO \end{array}\right]=\frac{1}{pathlength}{\left[\begin{array}{ccc} \varepsilon_{HbO}(\lambda_{1}) & \varepsilon_{HbR}(\lambda_{1}) & \varepsilon_{oxCCO}(\lambda_{1})\\ \varepsilon_{HbO}(\lambda_{2}) & \varepsilon_{HbR}(\lambda_{2}) & \varepsilon_{oxCCO}(\lambda_{2})\\ \vdots & \vdots & \vdots \\ \varepsilon_{HbO}(\lambda_{n}) & \varepsilon_{HbR}(\lambda_{n}) & \varepsilon_{oxCCO}(\lambda_{n}) \end{array}\right]}^{-1}\times \;\left[\begin{array}{c} \Delta A(\lambda_{1})\\ \Delta A(\lambda_{2})\\ \vdots \\ \Delta A(\lambda_{n}) \end{array}\right].$$

where pathlength is a product of the source/detector separation and the differential pathlength factor. The values of *ε* and DPF can be adjusted to determine the effect of uncertainties in these values on the resulting chromophore concentration change errors. The signal intensity can be given an SNR value in dB which is determined using:

(8) $$\text{SNR}=10\;{\text{log}}_{10}(\text{signa}l^{2}/{\text{noise}}^{2})$$

where ‘signal’ represents the signal intensity and ‘noise’ represents the noise added to the signal intensity. The percentage error between the true concentration change and the calculated concentration change (Δc) can then be determined via:

(9) %errorinΔc=((calculatedΔc−actualΔc)/actualΔc)×100.

It is prudent to examine the effect of errors in chromophore concentration changes on the reported polarity of the change. Most NIRS studies use an observed trend in the positive or negative direction of a change in chromophore concentration as evidence of effect [1]. In some cases this will meet the statistical threshold and reflect a true result, however for others the errors will be too great to say with any certainty that the polarity of the change is known. If the error in chromophore concentration change is greater than 100%, the true polarity of the change could be opposite to that reported.

## 3. Simulation input parameters

### 3.1. Wavelengths

**Fig. 1.**
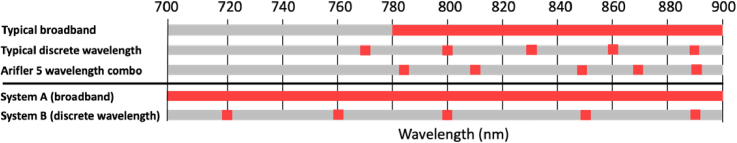
Illustration of typical broadband and discrete wavelength ranges taken from a recent review paper [3], the optimal combination from Arifler et al [21], and wavelengths used for diffusion simulations.

Conventionally, NIRS measurements of oxCCO have been made between 680-900 nm ^3^ as seen in Fig. 1. Below 650 nm, higher scattering and absorption effects limit the pathlength of photons in tissue [29]. Above this range, the absorption coefficient of water increases rapidly and dominates over smaller chromophore signals. Despite this, several studies have still used wavelengths outside of this rang [9,30–32].

In this study we sought to address how many wavelengths are required to resolve oxCCO alongside HbO and HbR with reasonable uncertainty. To this end, the error in chromophore concentration was calculated using the diffusion simulation for systems with increasing numbers of wavelengths. Two separate systems were simulated to explore different parameter effects - a broadband system with a wavelength range of 700-900 nm denoted System A and a five-wavelength system using 720, 760, 800, 850 and 890 nm denoted System B. These systems were used to explore the effects of uncertainties in extinction coefficient and DPF values, as well as the effects of changing source bandwidth and SNR. A summary of the typical wavelength ranges used to obtain measurements of oxCCO and those used for the simulations in this study is shown in Fig. 1.

**Fig. 2.**
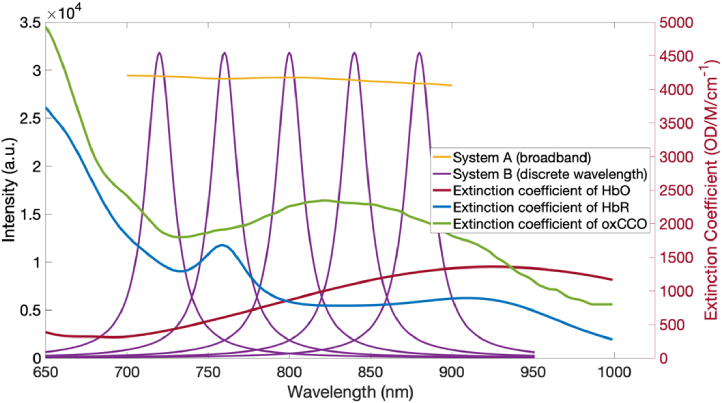
Graph of intensity spectra of System A and System B alongside the extinction coefficients of HbO, HbR and oxCCO. System B has central wavelengths 720, 760, 800, 840 and 890 nm each with a 20 nm bandwidth.

As well as the number of wavelengths, another potential source of inaccuracy is the bandwidth of the sources. Sources with a very large bandwidth can alter the data input required to the fitting algorithm and obscure the concentration signals. To assess the effect of source bandwidth on chromophore concentration accuracy, the bandwidth of each source in System B was changed from 1 to 50 nm and the resulting chromophore concentration error plotted. An example of the intensity spectra of System A and System B alongside the extinction coefficients of HbO, HbR and oxCCO is given in Fig. 2.

A method of mitigating against signal inaccuracies resulting from a large source bandwidth is to convolve the extinction coefficient spectra with the spectral shape of the source before fitting those spectra to the intensity signals. To explore the effect of performing a convolution on the accuracy of the chromophore concentration changes, simulations were conducted where the bandwidth of the five-wavelength system was changed, and the resulting concentration change errors both with and without a convolution were compared.

A crude estimate as to the quality of a matrix inversion is the condition number which, if low, demonstrates the matrix is well-conditioned and, if high, that it is ill-conditioned. As the modified Beer-Lambert equation involves performing a pseudo-inverse of the extinction coefficient matrix, the condition number can serve as an indicator for whether certain combinations might yield lower errors. One 1995 study found that the variance in chromophore concentration measurements could be minimised by selecting the optimal wavelengths according to their condition number [33]. Thus, all possible combinations of wavelengths were found for 3-50 wavelength sets across the 700-900 nm range, and sorted in order of descending 2-norm condition number generated by Matlab. The best combination and the 50th combination (to eliminate extreme outliers) were input to the diffusion simulation as the “best” and “worst” combinations. The chromophore concentration change errors for different numbers of measurement wavelengths for the “best” wavelength combinations were investigated with the diffusion simulation, with a 10% error in DPF and extinction coefficient applied.

### 3.2. Extinction coefficient and DPF

The values of extinction coefficient (*ε*) and DPF used across NIRS studies are often from different toolboxes or databases and have been derived from different sources. When comparing the most commonly used extinction coefficient values, they were found to differ by up to 10% within the commonly used NIRS range. One 2007 study aimed to estimate the errors in HbO and HbR concentration due to using different extinction coefficient datasets and found that the percentage difference in concentration output could be as high as 200% for small changes in HbO and HbR extinction coefficients [18].

The values of *ε* and DPF used to calculate the chromophore concentration changes were therefore changed by a varying percentage to observe the effect this had on chromophore quantification accuracy. DPF values were changed by 10% from the value generated by the broadband diffusion simulation. Values of *ε* as returned by the Homer2 software using values compiled by Prahl (oxCCO values originated from an animal study in 198 [34]) were also changed by 0 and 10%. Chromophore concentrations were calculated using each pair of values with respective errors applied. Chromophore concentrations were calculated using all possible positive and negative combinations of *ε* and DPF errors totaling four combinations, with the largest resulting concentration error taken as the value to plot for each error percentage.

The two most commonly used *ε* datasets were also used to directly compare the errors caused by their differences. A simulation was performed where the data from Homer2 (J.M. Schmitt) was used as input to the diffusion simulation, and a second dataset taken from the BORL website [35,36] used to calculate the chromophore concentration changes.

### 3.3. Concentration changes

A different concentration change was generated for each iteration of the diffusion simulation. The base concentrations of HbO, HbR and oxCCO in the slab were defined according to the literature [37]. The chromophore concentration change added to the base concentration was restricted to a specific range of variation, where each change was informed by the average of typical dynamic changes seen across recent clinical studies. Within this maximum range of variation, three separate concentration change limits were considered to determine the effect of the magnitude of the change on the associated chromophore concentration errors. A small, medium and large variation in concentration changes were investigated to represent typical changes in a neonate, adult and phantom. Within each of these ranges, the value taken to be the change in concentration was randomly chosen in the positive or negative direction using the MATLAB function randi, resulting in 100 different permutations averaged for each datapoint. This avoided biassing the simulation to a particular directional change.

Published works using NIRS to quantify changes in HbO, HbR and oxCCO from 2016-2022 were used to inform Model 1 and Model 2. Model 1 reflects the the average concentration changes observed in neonates during functional studies [38,39], and the average observed concentration changes from adults in a clinical setting who are undergoing paradigms designed to produce changes in systemic physiology were taken as the maximum range values for Model 2 [32,40–44]. Papers estimating changes in concentration of oxCCO from phantoms were used to inform Model 3 [19,25]. The base concentration values and the variation ranges for each of the three conditions are defined in Table 1.

### 3.4. SNR

As investigated in previous simulation work by Truong et al [19], knowledge of the SNR of system measurements is important for the interpretation of NIRS results. They found that the variance in concentration change measurements are minimised when the measurement system has an SNR equal to or greater than 50.

Therefore, the effect of applying different SNR to the output intensity values on chromophore concentration changes was explored, with the aim of validating the findings of the previous study. In the diffusion simulations, the SNR was varied by adding random Gaussian noise to the reflectance values using the MATLAB function awgn where the average fluctuation met the SNR threshold.

For real NIRS measurements, the source power and detector responsivity will also affect the achievable SNR and optimal wavelength combinations of each system. This has been explored in previous works [45,46]. Factors such as the maximum permissible exposure must also be considered to eliminate the possibility of burns or tissue damage. However, modelling all elements of hardware has not been attempted in this study, and the SNR values used are based on real values obtained at 30 mm source-detector separation for both broadband and traditional NIRS systems.

### 3.5. Diffusion method structure

The diffusion simulation was run multiple times with different parameters, however the main stages remained the same for each.

The number of wavelengths and their combination is first defined, and the simulation iterates through this number of wavelengths in the first loop. The wavelength range was 700-900 nm, and the combination of wavelengths used was evenly spread between this range. Most simulations were performed with 3-200 wavelengths, yielding N = 197 wavelengths sets. The extinction coefficient and DPF values were calculated by adding and subtracting the relevant percentage amount to be used in the final steps of the simulation. The percentage amounts of 0 and 10% were added to the DPF and *ε* values in the positive and negative direction yielding four sets of values.

Measured intensities were then simulated at the requisite number of wavelengths by iterating around the N sets of wavelengths. For each loop, a simulated concentration change loop was entered where the concentrations of HbO, HbR and oxCCO vary within a given range defined by the user.

The concentration changes of HbO, HbR and oxCCO are then changed over 100 iterations within this loop. These changes can be in the positive or negative direction within the given range, and change for each iteration. Within this loop, an SNR loop is entered to vary the noise for each iteration.

Within the SNR level loop, white Gaussian noise is added to the reflectance values at a given SNR value. This noise is added 100 separate times and the average resulting concentration value is taken.

Therefore, each data point for the concentration change error graphs is an average of 1,000 iterations of concentration change and noise. Each concentration change loop takes the average value from 100 noise iterations, and each wavelength number loop takes the average and standard deviation across the resulting 100 concentration change loops. Due to the large number of iterations, when the simulation is re-run using the same parameters, the results are consistent to within 1%.

**Fig. 3.**
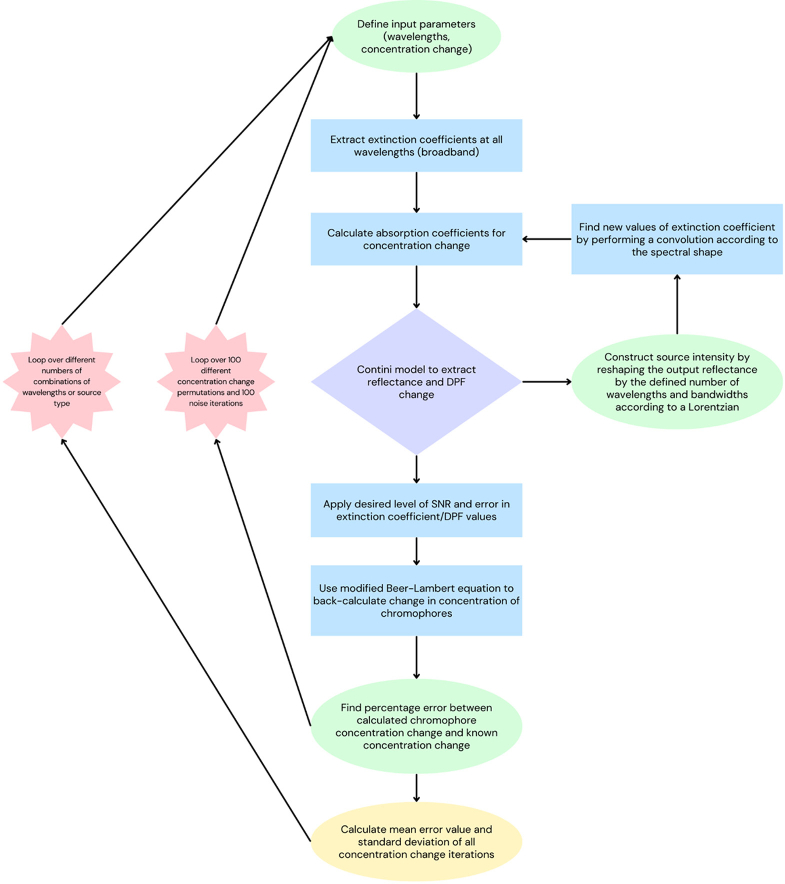
Flowchart showing main steps in the process.

A flow chart representing the main steps of the process is shown in Fig. 3. Parameters were changed according to the information in sections 3.1-3.4.

## 4. Results

### 4.1. Wavelengths

**Fig. 4.**
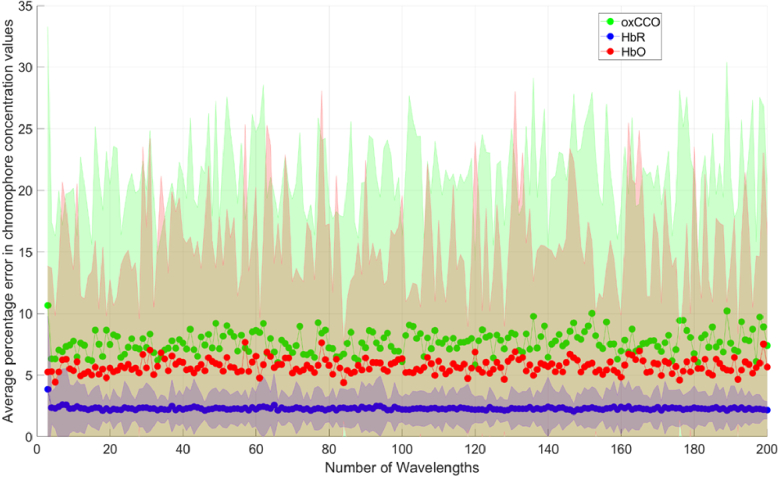
Percentage error in chromophore concentration changes against number of measurement wavelengths for ideal conditions (zero error in DPF and □ values, zero noise) for Model 1. Wavelength combinations are evenly spread across the 700-900 nm range. The shaded area denotes the standard deviation across a total of 1,000 iterations.

The average percentage errors in chromophore concentration changes with no error in DPF and extinction coefficients for systems with different numbers of measurement wavelengths are presented in Fig. 4. Each data point is an average of 300 concentration change permutations. Wavelength combinations were evenly spread across the 700-900 nm range.

The results in Fig. 4 show that, when measurement conditions are ideal (i.e. there is no uncertainty in *ε* and DPF values and the data is noiseless), measurement errors are not dependent on the number of measurement wavelengths and the means for each chromophore are all less than 10%. The error in chromophore concentration changes are non-zero as there are discrepancies between the diffusion approximation and the MBLL in terms of assumptions and fitting methods.

**Fig. 5.**
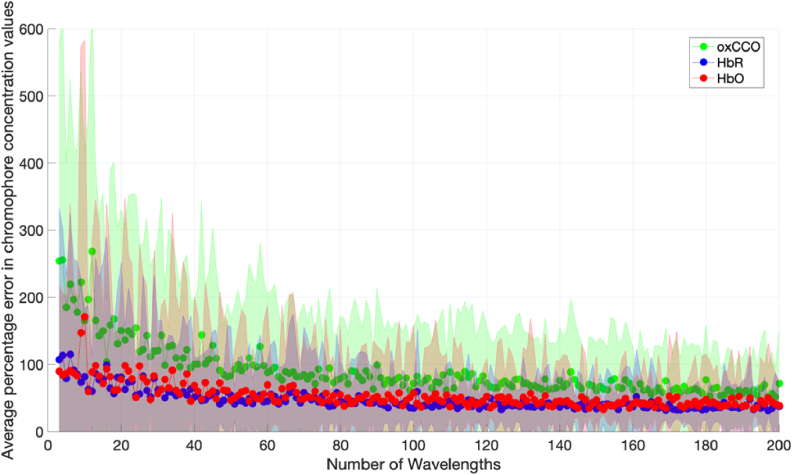
Percentage error in chromophore concentration changes against number of measurement wavelengths for 10% error in extinction coefficient and DPF (zero noise) for Model 1. Wavelength combinations are evenly spread across the 700-900 nm range. The shaded area denotes the standard deviation across a total of 1,000 iterations.

Errors in *ε* and DPF were then introduced to determine the magnitude of their effect on the error in chromophore concentration changes. Figure 5 shows the average percentage error in chromophore concentration changes when there is a 10% uncertainty in the DPF and extinction coefficients used to calculate concentration changes via the MBLL.

The mean percentage error in chromophore concentration following the introduction of a ten percent error in both DPF and *ε* is shown in Fig. 5. The plot demonstrates a steady reduction in chromophore concentration change error as the number of measurement wavelengths increases. oxCCO exhibits the most pronounced errors, exceeding 200% when utilising 3-10 measurement wavelengths and only falling below 100% with more than 80 measurement wavelengths.

**Fig. 6.**
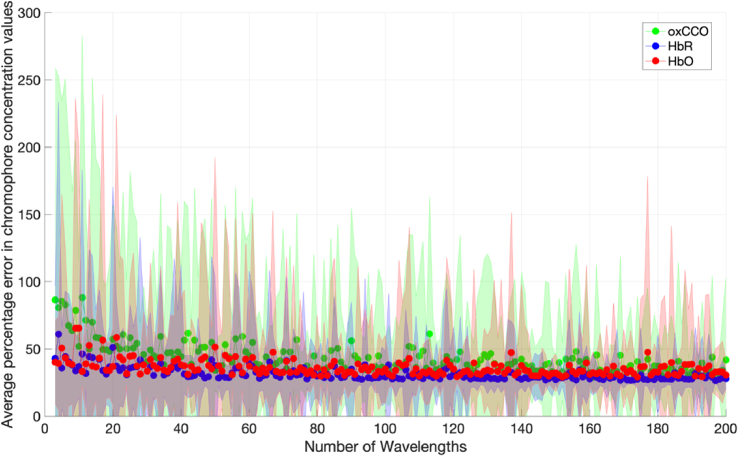
Graph of chromophore concentration errors against number of wavelengths with a 10% uncertainty in DPF and □, for Model 2.

**Fig. 7.**
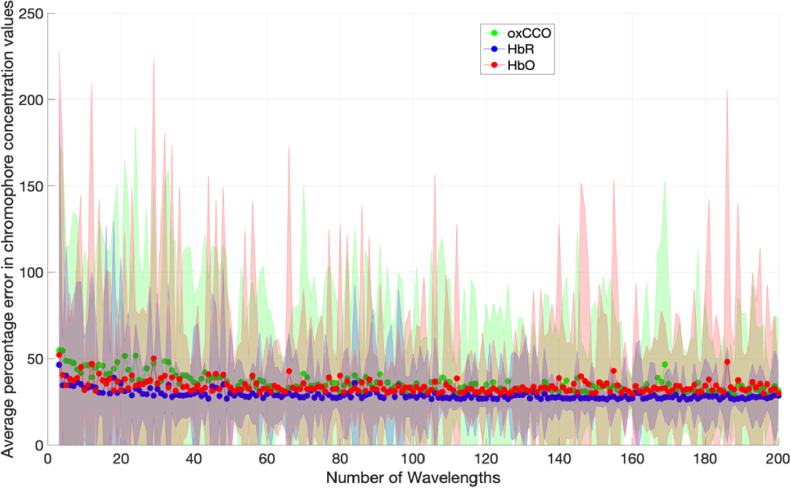
Graph of chromophore concentration errors against number of wavelengths with a 10% uncertainty in DPF and *ε*, for Model 3.

The concentration change boundaries were then varied as stipulated in Table 1 and used as input to the simulation. Figures 6 and 7 show the results of the simulations for each case.

Figures 6 and 7 both show decreasing errors with increasing number of measurement wavelengths. The errors are higher when the concentration changes are smaller as seen in Fig. 6. This result scales when compared to Fig. 4, which has the smallest concentration changes and the greatest concentration change errors. Figure 7 has the lowest errors and the greatest concentration changes.

The chromophore concentration change errors returned using the “best” and “worst” wavelength combinations according to their associated condition number were analysed but are not presented here (see Appendix Fig. 11 and Fig. 12). It was found that employing the optimal combinations does not significantly mitigate the error compared to utilising a set of wavelengths evenly distributed across the same spectrum range (as evidenced in Fig. 4). While leveraging the “best” wavelength combinations does not confer a distinct advantage, certain combinations yield notably larger chromophore concentration errors. Analysis of the “worst” wavelength combinations reveals that errors in oxCCO concentration changes are orders of magnitude higher for fewer wavelengths and persistently exceed 100% across the 3-50 wavelength range. Notably, many of these “worst” combinations comprise wavelengths closely clustered together. Previous studies, such as Arifler et al [21], have observed that utilising evenly distributed wavelength combinations tends to minimise errors, a finding that aligns with the results presented here.

**Fig. 8.**
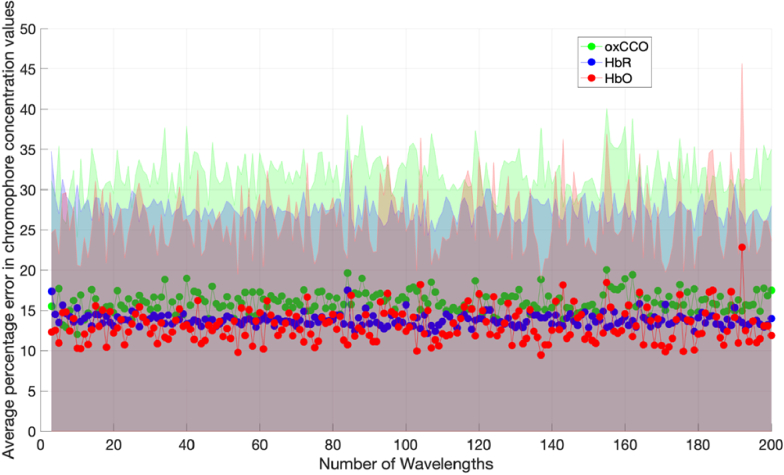
Errors in chromophore concentration changes against number of measurement wavelengths using two different *ε* datasets on Model 1. Wavelength combinations are evenly spread across the 700-900 nm range. Input values of *ε* to the simulation were generated by the Homer2 software using values compiled by Prahl (oxCCO values originated from an animal study in 198 [34]). Values of *ε* used to calculate concentration changes via the MBLL were taken from Cope 199 [47].

Extinction coefficient values from one dataset were used to construct the inputs to the diffusion equation, and a second dataset was used to calculate the concentration values using the MBLL. The errors in the chromophore concentration changes resulting from using different □ datasets for the forwards and backwards calculations in each simulation are shown in Fig. 8.

The chromophore concentration change errors in Fig. 8 are higher than those seen in Fig. 4, and indicate that the baseline error for ideal conditions may be around 15% (as seen in Fig. 8) due to inconsistencies in real □ datasets.

### 4.2. Bandwidth

**Fig. 9.**
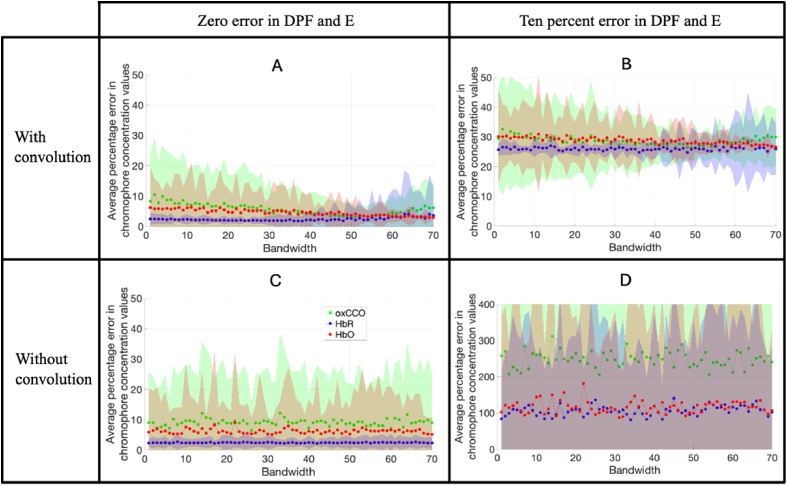
The percentage error in chromophore concentration changes for Model 1 plotted against spectral bandwidth (in nm) for a five-wavelength system (System B) for zero and 10% uncertainties in DPF and □, and with and without a convolution applied (no noise) Graph D has a different y-axis to A, B and C.

The diffusion model was used to illustrate the dependency of the error in chromophore concentration changes on bandwidth for System B (described in section 3.1). The bandwidth was varied between 1 nm and 70 nm, assuming either zero or ten percent uncertainty in both DPF and □. Results were obtained both with and without adjusting the extinction coefficient values by convolving with the corresponding spectral profile of the source. The results are shown in Fig. 9.

When the convolution is not applied, there is no apparent correlation between chromophore concentration error and bandwidth. When DPF and □ are known precisely, the uncertainties in the changes of all three chromophores are consistently less than ten percent, irrespective of the bandwidth (Fig. 9(C)). However, for a ten percent uncertainty in DPF and □, the concentration change errors are more than 20 times greater without convolution (Fig. 9(D)).

When a convolution is applied, however, the chromophore concentration errors display a notable dependency on bandwidth, especially that of oxCCO. The errors slightly *decrease* with increasing bandwidth up to about 60 nm, after which the error increases, and rapidly in the case of oxCCO (Fig. 9(A),(B)).

Introducing the ten percent uncertainty in DPF and □ increases the errors at all bandwidths (around 100% for HbO and HbR and >200% for oxCCO); the increase is far less than when the convolution was not applied.

### 4.3. SNR

**Fig. 10.**
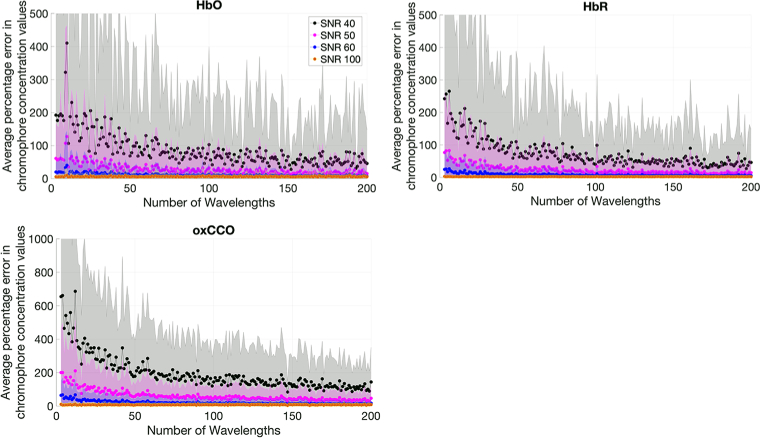
Graphs comparing the errors in concentration change of HbO, HbR and oxCCO of systems with different wavelengths and varying SNR as calculated using the diffusion simulation using Model 1. Standard deviation across 300 concentration changes and 300 noise iterations is shown as a shaded error bar.

**Fig. 12.**
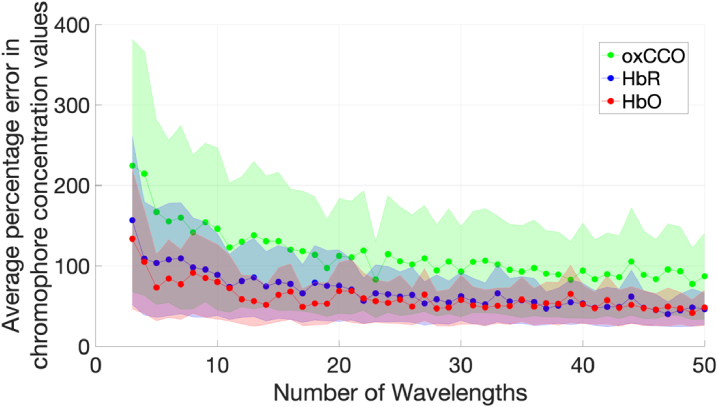
Graph of percentage error chromophore concentration changes against number of measurement wavelengths for the ‘best’ wavelength combinations sorted by condition number.

As illustrated in Fig. 10, increasing the SNR of the intensity measurement led to a reduction in chromophore concentration errors. The average percentage chromophore concentration error against the number of measurement wavelengths is plotted for each chromophore at each SNR level. The errors across the whole range of wavelengths for the hemoglobins reduce to below 100% at an SNR of 50, but require an SNR of 60 to reduce below 100% for oxCCO across the entire range. These outcomes, derived from the diffusion simulation, are consistent with data from Truong et al [19], who observed a comparable trend. Our findings corroborate their assertion that an SNR of 60 represents the minimum threshold for precise NIRS-derived concentration change assessments at low numbers of wavelengths.

## 5. Discussion

The results from the diffusion simulation analysis reveal that, for systems with small numbers of wavelengths, errors in chromophore concentration changes can be very large. The magnitude of the error depends on the number of wavelengths, the bandwidth of the sources and the error in DPF and extinction coefficient values used.

For most clinical studies the percentage error on measurements must fall far below 100% to be considered significant. For the purposes of discussion, we suggest that in order to be confident in both the direction of the concentration changes of HbO, HbR and oxCCO and their relative magnitudes, a percentage error below 50% must be achieved.

In general, when there is zero uncertainty in DPF and *ε*, measurement errors fall below the acceptable level of 50%. This suggests that efforts to more accurately determine these values *in vivo* could aid in reducing errors for all systems.

This study sought to provide information on the effects of the number of wavelengths and characteristics related to the optical sources. While these initial results using a slab geometry provide relevant information, reproducing these results with Monte-Carlo simulations and anatomically appropriate head models would be valuable.

### 5.1. Errors are dependent on number of measurement wavelengths

As seen in Fig. 4, if the number of wavelengths used is less than 50 and the error in the DPF and *ε* is 10%, the error in chromophore concentration change can be greater than 50%. This is consistent with previous work indicating that small uncertainties in extinction coefficients could result in large errors in NIRS-derived chromophore concentration change [18].

Assuming uncertainties in DPF and *ε* of 10%, a system with an SNR greater than 40 with at least 50 measurement wavelengths is required for all chromophore concentration change errors to be less than 100%, which is in agreement with other simulation and phantom studies [19]. This points towards broadband systems being the optimal choice for conditions where DPF cannot be accurately measured. However, if the DPF can be simultaneously measured alongside the chromophore concentration changes, the threshold conditions required for less than 100% chromophore concentration change errors are different. Chromophore concentration change accuracy can be improved by using more accurate DPF values, which can be estimated [48] or effectively measured during experiments using other techniques [25,49,50]. If the DPF uncertainty is minimised and there is a conservative 10% error in *ε*, a system with 40 wavelengths is required for measurement errors to likely be below 100%. It is worth noting that the percentage uncertainties in DPF and *ε* were applied in combination in the positive and negative direction, with the greatest error resulting from the four combinations taken as the value for analysis. This could result in the errors being exaggerated as, in practice, the uncertainties may be different.

We have shown that, in order to obtain accurate results (< 30% error) with a small number of wavelengths, highly reliable input data is required. If the extinction coefficients of HbO, HbR and oxCCO were known with greater certainty, and the DPF simultaneously measured alongside the chromophore concentration changes, there appears to be very little dependence on the number of measurement wavelengths on their concentration change errors for signals with high SNR, providing a convolution is performed for LED systems. While the effect of a large source bandwidth can be minimised by performing a convolution, only by decreasing the uncertainties in DPF and *ε* are the measurement errors well below 30%. Therefore, based on these findings, there is renewed motivation for updated measurements of these extinction coefficients in realistic environments, which has been highlighted by several groups previously [18,51]. Additionally, if the uncertainty in the measured extinction coefficients were to be provided, this could allow more precise estimation of the likely chromophore concentration change errors for future NIRS studies.

### 5.2. Wavelength combinations do not significantly affect errors

Previous Monte-Carlo simulation work has indicated that excluding the wavelength pair 780 and 830 nm can improve haemoglobin resolution [52] as data collected by these wavelengths suffer from greater noise contamination [53]. Efforts to identify the optimal wavelength combination based on their condition number in this study have not produced a significant improvement in the accuracy of chromophore concentration changes. This outcome is likely attributable to the application of uniform noise levels to each signal within the simulations, leading to discrepancies when compared to in vivo data. Another possible explanation is that most wavelength combinations, provided that some wavelengths are reasonably spaced apart, yield a low condition number. Consequently, only unrealistic combinations, such as multiple wavelengths clustered closely together, produce a high condition number, which is associated with greater errors. When considering the optimal wavelength combination for extracting simultaneous concentration changes of HbO, HbR and oxCCO, provided the wavelengths of choice are spread relatively evenly across the wavelength range used, there does not appear to be a benefit in attempting to choose a combination that returns a lower condition number. This finding is in conflict with other studies stating that utilising the wavelength combination with the lowest condition number will minimise associated error [29].

### 5.3. LED systems require consideration of their spectral range to observe changes in all chromophores

As seen in the comparison of graphs illustrating the percentage error in chromophore concentration changes against the source bandwidth in Fig. 9, if the measurement parameter errors are small and the bandwidth accounted for via a convolution with the extinction spectra, the errors in chromophore concentration change can be reduced to below 100%. Results appear to suggest that regardless of the magnitude of the source bandwidth, the chromophore concentration change errors are reduced by performing a convolution. Convolution of the collected intensity with the spectral shape has been performed on the laser diodes in the processing pipeline of a commercial time-domain NIRS instrument [54] and appears to be standard for the use of NIRS in the food quality assessment industry. However it has not been commonplace for studies using LED-based NIRS devices thus far. Our results show, even more consequentially, that the concentration change in both hemoglobins and oxCCO might not be able to be accurately determined by LED systems without performing a convolution.

The effect of a convolution decreasing the measurement errors with increasing bandwidth is likely due to a trade-off between the advantage of incorporating a greater range of wavelengths when the bandwidth is increased (i.e. increasing the spectral information within the discrete measurements from each of the five sources, providing the bandwidth does not exceeds the interval between the wavelengths) and the disadvantage of blurring the spectral features in the extinction coefficient spectra. Consequently the effect on the estimates of HbO is smaller because of the relative flatness of the extinction coefficient spectrum over the 700 - 900 nm range, and convolution has less of an effect.

### 5.4. Errors are dependent on the expected magnitude of concentration change

The errors in chromophore concentration change also seem to be dependent on the expected magnitude of the concentration change, which is difficult to predict for most studies. Figures 6 and 7 show that the greater the magnitude of the concentration change, the lower the error in chromophore concentration change as calculated via the diffusion simulation. All three chromophore concentration changes are realistic and within the range seen in recent NIRS studies. This suggests that the NIRS system design should be matched to the application; if the concentration changes expected are large (e.g. clinical scenarios), a system with a low number of wavelengths can be tolerated, but for cases with small concentration changes (e.g. functional studies) a higher number of wavelengths will be required.

## 6. Conclusion

This work has explored the impact of NIRS system hardware choice and uncertainties in measurement parameters on the accuracy of calculated chromophore concentration changes. A diffusion simulation was constructed to simulate photon migration through a finite slab, where the input parameters were changed to determine their impact on the accuracy of concentration changes of HbO, HbR and oxCCO. Systems which use fewer than 50 measurement wavelengths and with larger bandwidths unaccounted for via a convolution with the extinction spectra suffer from greater inaccuracies in results. Such systems also may not be capable of accurately reconstructing the oxCCO signal without performing a convolution according to their spectral shape.

When there are errors of 10% in the DPF and ε values used, the measurement errors can be over 100% for HbO and HbR, and over 200% for oxCCO in in-vivo measurements. Therefore, when constructing a device to measure changes in oxCCO, the source should be fully characterised and any required adjustments to the processing pipeline implemented. If using LED sources, a convolution should be performed to adjust the values of extinction coefficient used. The scattering changes should be measured either through the use of frequency domain [55] or time-resolved [56] NIRS systems, or using a spectral fitting method with a broadband NIRS syste [57], and the DPF calculated for each experiment. Extinction coefficient values, especially those for oxCCO, could benefit from an updated measurement in realistic environments to ensure accuracy.

There exists potential for systems with any number of measurement wavelengths and bandwidth to accurately measure HbO, HbR and oxCCO simultaneously provided there are appropriate hardware and processing adjustments. It is important for these issues to be addressed in order to have higher confidence in NIRS-derived oxCCO signals and for studies measuring oxCCO to have a greater impact. In order to estimate the expected concentration changes associated with a certain population and activation paradigm, it is recommended to consult previous literature and compare with studies which have conducted a similar experiment with a broadband system. For phantom experiments, where the expected concentration changes are larger, a system with as few as 3 measurement wavelengths could be sufficient for accurate results as seen in Fig. 7. For human studies where the expected concentration changes are smaller, a system with more than 8 wavelengths is required for the concentration change errors to fall below 50% (see Fig. 6) and obtain reliable measurements.

## Figures and Tables

**Fig. 11.**
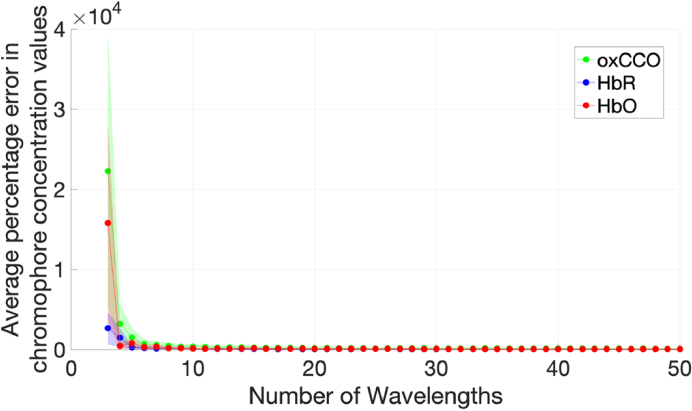
Graph of percentage error chromophore concentration changes against number of measurement wavelengths for the ‘worst’ wavelength combinations sorted by condition number.

## Data Availability

Data underlying the results presented in this paper are not publicly available at this time but may be obtained from the authors upon reasonable request.
